# Evaluation of drugs used in chronic heart failure at tertiary care centre: a hospital based study

**DOI:** 10.15171/jcvtr.2019.15

**Published:** 2019-06-30

**Authors:** Rinku Ghimire, Sahadeb Prasad Dhungana

**Affiliations:** ^1^Department of Pharmacology, Nobel Medical College Teaching Hospital, Nepal; ^2^Cardiology Unit, Department of Internal Medicine, Nobel Medical College Teaching Hospital, Nepal

**Keywords:** Chronic Heart Failure, Drugs, Evaluation

## Abstract

***Introduction:*** There is lack of data on pattern of use of drugs in patients with chronic heart failure (CHF) from Nepalese population. This study was conducted to explore the trends of evidence based medications used for CHF in our population.

***Methods:*** This is a cross-sectional study on 200 consecutive patients with New York Heart Association (NYHA) class II to IV symptoms of CHF who attended cardiology clinic or admitted from September 2017 to August 2018 at Nobel Medical College Teaching Hospital, Biratnagar, Nepal.

***Results:*** Mean age of patients was 54 (range 15-90) years. Ischemic cardiomyopathy, rheumatic heart disease, dilated cardiomyopathy, hypertensive heart disease, peripartum cardiomyopathy were common etiologies of CHF. Analysis of drugs used in CHF revealed that 85% patients were prescribed diuretics, 58.5% angiotensin-converting enzyme inhibitors (ACEIs) or angiotensin receptor blockers (ARBs), 53% mineralocorticoid receptor antagonists (MRAs), 38% beta-blockers (BBs) and 24% digoxin. Digoxin was mainly used as add on therapy for patients with atrial fibrillation (24% of all patients). Antithrombotics (warfarin or aspirin), inotropic agents (dopamine, dobutamine or noradrenaline), antiarrhythmic agent (amiodarone) and nitrates (intravenous glyceryl trinitrate or oral isosorbide dinitrate) were prescribed for 48%, 28%, 5% and 6% patients respectively. All CHF patients with preserved or mid-range ejection fraction (25% of all patients) were prescribed diuretics along with antihypertensive drugs for hypertensive patients.

***Conclusion:*** CHF is associated with significant morbidity and mortality due to associated co-morbidities and underuse of proven therapy like BBs, ACEIs or ARBs and MRAs. Careful attention to optimization of different drugs therapy in patients with CHF may help to improve patient outcomes.

## Introduction

Chronic heart failure (CHF) is a common cardiac problem with significant morbidity and mortality. It is a clinical syndrome caused by various cardiac conditions that impair the ability of the ventricle to fill with or eject blood.^1^

The goals of treatment of CHF are the reduction of symptoms, minimization of the number of hospitalizations and prevention of premature death. The mainstay of treatment is lifestyle modifications and pharmacologic therapy. Implantable devices and surgery may be needed in selective patients. Angiotensin-converting enzyme inhibitors (ACEIs) or angiotensin receptor blockers (ARBs), beta-blockers (BBs) and mineralocorticoid receptor antagonists (MRA) have been shown to improve clinical condition and survival of patients with CHF with reduced ejection fraction (HFrEF).^2^ Diuretic therapy helps to reduce cardiac load with improvement in left ventricular (LV) function.^3^ Mortality and morbidity after symptomatic CHF have remained high although variable which could be due to differences in severity of disease and appropriate use of evidence-based treatment.

The number of patients with CHF is increasing in low-income countries like Nepal, as result of the adoption of western type lifestyles, leading to an increased number of risk factors, aging of the population and still a high burden of rheumatic heart disease. Due to the lack of data on the evidence-based treatment used in CHF, this study was conducted to explore the trends of medications used for CHF in the Nepalese population.

## Materials and Methods

This is a cross-sectional hospital-based study. Total of 200 consecutive patients with a diagnosis of CHF who attended the cardiac clinic or admitted from September 2017 to August 2018 at cardiology unit, department of internal medicine of Nobel Medical College teaching hospital, Nepal were included in the study. The aim was to evaluate the current trends of the use of evidence-based drugs in patients with CHF. All patients with a diagnosis of CHF with reduced or preserved ejection fraction based on Framingham Criteria and echocardiographic assessment was included.

Patients with CHF were categorized as having heart failure with preserved ejection fraction (HFpEF) (EF ≥50%), heart failure with reduced ejection fraction (HFrEF) (EF <40%), heart failure with mid-range ejection fraction (HFmrEF) (EF 40%–49%), based on the recent European Society of Cardiology guidelines. Inclusion criteria were: 1. Age ≥15 years, 2. Patients attending the cardiac clinic with a diagnosis of CHF 3. Patients admitted to the cardiology unit with a diagnosis of acute decompensation of heart failure (ADHF). Exclusion criteria were (1) Acute de novo heart failure after acute coronary syndrome or acute myocarditis, (2) Asymptomatic patients with echocardiographic evidence of LV dysfunction.

Demographic and clinical variables were noted during enrollment which included age, gender, and underlying etiology of CHF, co-morbidities, and medications used by the patient. The serum electrolytes, renal function tests, electrocardiographic and echocardiographic parameters were reviewed from the case record of patients.

### Statistical analysis

Data were entered in Microsoft Excel 2007 and converted into IBM Corp. Released in 2011. IBM SPSS Statistics for Windows, Version 20.0. Armonk, NY: IBM Corp. Continuous and categorical variables were presented as mean, percentage and interquartile range wherever found necessary. The tabular presentation was made for necessary variables.

## Results

Two hundred consecutive patients with a diagnosis of CHF were included in the study. There were 110 (55%) women and 90 (45%) men. The mean age of patients was 54 (range 15-90) years. Seventy-four (37%) patients were cigarette smokers and 28 (14%) had a significant history of alcohol consumption. About one third 64 (32%) of patients were hypertensive and 40 (20%) were diagnosed to have diabetes mellitus. Baseline characteristics of patients with CHF are summarized in Table 1. Among the underlying co-morbidities, 120 (60%) had anemia, 60 (30%) had underlying coronary artery disease, 30 (15%) had acute kidney injury, 20 (10%) had chronic kidney disease, 10 (5%) were using treatment for chronic obstructive airway disease. Co-morbidities and electrolytes imbalance of patients with CHF have been mentioned in Table 2.

**Table 1:** Baseline characteristics of patients with chronic heart failure (n=200)

Age in year (mean and range)	54 (16-90)
Female	110 (55%)
Male	90 (45%)
Heart rate in bpm (mean and range)	90 (60-160)
Tachycardia (Heart rate >100 bpm)	60 (30%)
Blood pressure in mm Hg (mean and range)	
Systolic	112 (60-210)
Diastolic	74 (40-100)
Presenting symptoms	
Dyspnea (NYHA class) IV	120 (60%)
III	44 (22%)
II	36 (18%)
Edema	156 (78%)
HFrEF	150 (75%)
Stable (outpatient)	110 (73.3%)
ADHF (admitted)	40 (26.6%)
HFpEF	38 (19%)
HFmrEF	12 (6%)

Abbreviations: NYHA, New York Heart Association; BPM, beat per minute; ADHF, acute decompensated heart failure; HFrEF, heart failure with reduced ejection fraction; HFpEF, heart failure with preserved ejection fraction; HFmrEF, heart failure with a mid-range ejection fraction.

**Table 2:** Co-morbidities and electrolytes imbalance of patients with chronic heart failure (n=200)

**Co-morbidities**	**HFrEF (n=150)**	**HFpEF (n=38)**	**HFmrEF (n=12)**
Anemia	112 (74.6%)	6 (15.7%)	2 (16.6%)
Hypertension	32 (21%)	28 (73.6%)	4 (33.3%)
Coronary artery disease	48 (32%)	10(26.3%)	2 (16.6%)
Type 2 diabetes mellitus	31(20.6%)	9 (23.6%)	0
Chronic kidney disease	16 (10.6%)	4 (10.5%)	0
Hypothyroidism	9 (6%)	1 (2.6%)	0
Chronic obstructive pulmonary disease	10(6.6%)	-	-
Stroke	6 (4%)	-	-
Hyperthyroidism	3(2%)	-	-
Pulmonary embolism	3 (2%)	-	-
Electrolytes imbalance Hyponatremia (<135 mEp/L) Hypernatremia (>145 mEq/L) Hypokalemia (< 3.5 mEq/L) Hyperkalemia (> 5 mEq/L)	58(38.6%) 14(9.3%) 11(7.3%) 16(10.6%)	4 (10.5%) - 4 (10.5%) 2 (5.2%)	- - 1 (8.3%) -

Abbreviations: HFrEF: heart failure with reduced ejection fraction; HFpEF: heart failure with preserved ejection fraction, HFmrEF: heart failure with a mid-range ejection fraction.

Ischemic cardiomyopathy 50 (25%), rheumatic heart disease 44 (22%), dilated cardiomyopathy 20(10%), hypertensive heart disease 16(8%), peripartum cardiomyopathy 12 (6%), cor pulmonale 12(6%), uremic cardiomyopathy (patients with chronic kidney disease) 12 (6%) were the common etiologies for CHF. Other causes of CHF included congenital heart diseases 10(5%), alcohol-related cardiomyopathy 10 (5%), pericardial diseases 6 (3%), sclerodegenerative aortic stenosis 4 (2%), tachycardia-induced cardiomyopathy 4 (2%).

As mentioned in Table 3, different diuretics agents were prescribed to 85% patients. MRAs were given to 106 (53%), ACEIs to 96 (48%) or ARBs to 21(10.5%) patients. Forty-eight patients (24%) were given digoxin. Antithrombotics (warfarin or aspirin), inotropic agents (dopamine, dobutamine or noradrenaline), antiarrhythmic agent (amiodarone), nitrates (intravenous glyceryl trinitrate or oral isosorbide dinitrate) were prescribed to 96 (48%), 56 (28%), 10 (5%), 12 (6%) patients respectively. Different drugs combination used in patients with CHF has been illustrated in Table 4. Electrocardiographic and echocardiographic findings are summarized in Table 5.

**Table 3:** Individual drugs used in patients with chronic heart failure (n=200)

**Drug class**	**Drug**	**HFrEF (n=150)**	**HFpEF (n=38)**	**HFmrEF (n=12)**
Diuretics	Furosemide Torsemide Metolazone Indapamide Hydrochlorthiazide	88 (58.6%) 28 (18.6%) 2 (1.3%) 4 (2.6%) 1 (0.6%)	26 (68.4%) 8 (21%) 2 (5.2%) - 1 (2.6%)	6 (50%) 4 (33.3%) -
MRAs	Spironolactone Epleronone	64 (42.6%) 10 (5%)	22 (57.8%) 0	10 (83.3%)
ACEIs	Enalapril Ramipril	40 (26.6%) 30 (20%)	9 (23.6%) 5 (13.1%)	7 (58.3%) 5 (41.6%)
ARBs	Losartan Telmisertan Olmesertan	4 (2.6%) 8 (5.3%) 3 (2%)	4(10.5%) - -	2 (16.6%)
BBs	Metoprolol Carvedilol Bisoprolol Nebivolol	52 (34.6%) 18 (12%) 4 (2.6%) 2 (1.3%)	- - - -	
Glycosides	Digoxin	40 (26.6%)	2(5.2%)	6 (50%)
Antithrombotics	Aspirin Warfarin	62(41.3%) 26 (17.3%)	3 (7.8%) -	5 (41.6%)
Inotropic agents	Dobutamine	28 (14%)	-	
	Dopamine	22 (11%)	-	
	Noradrenaline	6 (3%)	-	
Nitrates	GTN Isosorbide dinitrate	8 (4%) 4 (2%)	- -	
Antiarrhythmics	Amiodarone	10 (5%)	-	

Abbreviations: ACEIs, Angiotensin-converting enzyme inhibitors; ARBs, Angiotensin receptor blockers; BBs, Beta blockers; MRAs, Mineralocorticoid receptor antagonists; GTN, Glyceryl trinitrate; HFrEF, heart failure with reduced ejection fraction; HFpEF, heart failure with preserved ejection fraction; HFmrEF, heart failure with a mid-range ejection fraction.

**Table 4:** Different drugs combination used in patients with chronic heart failure (n=200)

**Drugs**	**HFrEF (n=150)**	**HFpEF (n=38)**	**HFmrEF (n=12)**
Diuretics (only)	4 (2.6%)	25 (65.7%)	7 (58.3%)
Diuretics + ACEIs/ARBs	46 (30.6%)	13 (34.2%)	5 (41.6%)
Diuretics + ACEIs + BBs	40 (20%)	-	
Diuretics + BBs	32 (16%)	-	
Diuretics +ACEIs + BBs + MRAs	28 (14%)	-	

Abbreviations: ACEIs, Angiotensin-converting enzyme inhibitors; ARBs, Angiotensin receptor blockers; BBs, Beta blockers; MRAs, Mineralocorticoid receptor antagonists; HFrEF, heart failure with reduced ejection fraction; HFpEF, heart failure with preserved ejection fraction; HFmrEF, heart failure with a mid-range ejection fraction.

**Table 5:** Electrocardiographic and Echocardiographic findings of patients with chronic heart failure (n=200)

**Electrocardiographic findings**		**Echocardiographic findings**	
Heart rate (BPM)		LVEF (%)	
Normal (60-100)	140 (70%)	<40	150 (75%)
Tachycardia (>100)	60 (30%)	≥50	38 (19%)
		40-49	12 (6%)
Rhythm		PASP	
Sinus	152 (76%)	Raised (≥ 40 mmHg)	170 (85%)
AF	48 (24%)	Normal (<40 mmHg)	30 (15%)
LVH	40 (20%)	RV dysfunction	40 (20%)
LBBB	14 (7%)	MR	84 (42%)
RBBB	8 (4%)	LVDD	132 (66%)

Abbreviations: AF, Atrial fibrillation; BPM, beat per minute; RBBB, right bundle branch block; LBBB, Left bundle branch block; LVH, Left ventricular hypertrophy; LVEF, Left ventricular ejection fraction; PASP, Pulmonary artery systolic pressure; RV, Right ventricle; MR, Mitral regurgitation; LVDD, Left ventricular diastolic dysfunction.

## Discussion

This study evaluated the patterns of use of different evidence-based therapy in patients with CHF. There have been advances in the drug treatment of CHF over the past decades. The aims of CHF treatment are the reduction of symptoms and improvement of survival. Treatment should be focused on minimizing the detrimental effects of neurohormonal compensatory mechanisms.

Sodium and water retention is common in CHF and diuretics are prescribed for patients with pulmonary or peripheral congestion.^4^ Diuretic therapy results in favorable effects with a reduction in cardiac preload and afterload with improvement in LV function.^5^ Eighty-five percent of our patients were prescribed diuretics at the time of admission except those who had hypotension or cardiogenic shock requiring inotropic support. The majority received loop diuretics. ACEIs or ARBs, BBs, and MRAs have been documented to improve clinical status and survival of patients with CHF.^2^ The proven effects of ACE inhibition to prolong survival support their use as first-line agents in the management of CHF.^6^ Only 58.8% of our patients were prescribed ACEIs or ARBs owing to a lower range of blood pressure or fear of worsening renal function. Similarly, only 38% of patients were started on BBs due to fear of decompensation or failure to achieve a euvolemic state. We noted underuse of disease-modifying drugs such as BBs, ACEI or ARBs and no prescription of combined hydralazine and isosorbide probably due to unavailability of this drug in our country. Although the use of BBs, ACEIs or ARBs have improved than previous years as a study done in Kathmandu valley by Baskota et al ^7^ in 2006 showed even more restricted use of these therapies. Serum aldosterone level is found to be elevated in patients on ACE inhibitor and may contribute to the worsening of HF. Spironolactone is a competitive antagonist of aldosterone and shows beneficial effects in patients already treated with ACE inhibitor.^8^ In our study, the use of spironolactone was more liberal since 53% of our patients were prescribed spironolactone.

The Digitalis Investigation Group showed no mortality benefit of digoxin in CHF.^9^ However, a digoxin withdrawal study has shown to improves exercise capacity and the need for the hospital admission.^10^ Digoxin has been prescribed commonly to control the ventricular rate in patients with CHF and AF. It would be reasonable to use digoxin in symptomatic patients despite an adequate dose of diuretics, ACE inhibitors, and BBs. In our study, digoxin has been used mainly in patients with AF (24%) with CHF as add on therapy without monitoring of serum level.

Based on the pattern of dysfunction (HFrEF, HFmrEF or HFpEF), there were differences in the frequency of use of ACE inhibitors/ARBs, BBs and MRAs, being higher in HFrEF. This can be explained by the presence of good evidence supporting the efficacy of these drugs in patients with HFrEF.

No good evidence exists for the benefit of diuretics, ACE inhibitors,^11^ BBs, MRAs or calcium antagonists in patients of HFpEF. Diuretics are often used to reduce and prevent fluid overload. Similarly, all our patients have prescribed loop diuretic agents along with antihypertensive drugs for hypertensive patients.

HFmrEF might be managed in the same way as HFpEF because of limited evidence on it.^12^ Our small number of patients with HFmrEF were managed in the same way as HFpEF. Although some studies demonstrated that BBs or ACEI/ARBs significantly improves the prognosis for patients with HFmrEF.^13,14^

The prevalence of AF in patients with HF ranges from 10 to 30%^15^ and has been observed to increase with increasing severity of HF.^16^ Twenty four percent of our patients with CHF had AF. HF increases the risk of thromboembolism in patients with AF. Thus, antithrombotic therapy with either aspirin or a vitamin K antagonist warfarin is needed. The choice is made based on the presence of risk factors for thromboembolism, the risk of bleeding manifestations and patient preferences.^17^ In our study, only around half (54%) of all eligible patients had been prescribed warfarin indicating the marked underuse of anticoagulant which might be due to difficulty in monitoring the monthly INR or reluctance on part of the physician. Some of the possible reasons for underuse of standard medications for CHF in our study may include poor awareness among physicians, high cost of multidrug therapy and the late presentation and severity of CHF which may prohibit the use of all proven therapies. Many physicians are still not comfortable commencing BBs in severely ill patients with CHF.

Acute decompensated heart failure (ADHF) is a common problem with limited treatment options. There is a lack of consistent benefit with diuretics, vasodilators, and inotropes for ADHF although they are used frequently to stabilize the acute symptoms.^18^ Around 20% of our patients who presented with ADHF were prescribed different inotropes or nitrates with an attempt to initial stabilization.

There are some limitations to this study. This was a hospital-based study in the limited number of patients who mainly represents severely symptomatic patients. Diagnosis of ischemic heart disease was based on history, risk factors, wall motion abnormality in echocardiography and may not be accurate because coronary angiography was not done in all cases.

CHF is a common problem and associated with significant morbidity and mortality because of associated co-morbidities and underuse of proven therapy like BBs, ACEIs or ARBs and MRAs. Diuretics are necessary for patients with evidence of pulmonary or peripheral congestion. If tolerated, ACEIs and BBs reduce mortality and can prevent the progression of symptoms and the need for hospitalizations. Low dose MRAs improves survival in patients with CHF with ongoing symptoms despite standard therapy. Careful attention to the optimization of different drugs therapy in patients with CHF may help to improve patient outcomes.

## Ethical approval

Ethical approval was obtained from the institutional review committee (IRC) prior to starting the study (IRC NMCTH 205/2018).

## Competing interests

All authors declare no competing financial interests exist.
